# Electronic health record nested pragmatic randomized controlled trial of a reminder system for serum lithium level monitoring in patients with mood disorder: KONOTORI study protocol

**DOI:** 10.1186/s13063-019-3847-9

**Published:** 2019-12-11

**Authors:** Tomotsugu Seki, Morio Aki, Hirotsugu Kawashima, Tomotaka Miki, Shiro Tanaka, Koji Kawakami, Toshi A. Furukawa

**Affiliations:** 1 Department of Pharmacoepidemiology, Graduate School of Medicine/School of Public Health, Kyoto University, Kyoto, Japan; 2 Department of Psychiatry, Toyooka Hospital, Toyooka, Hyogo Japan; 3 Department of Psychiatry, Kyoto University Hospital, Kyoto, Japan; 4 Department of Clinical Biostatistics, Graduate School of Medicine, Kyoto University, Kyoto, Japan; 5 Department of Health Promotion and Human Behavior, Graduate School of Medicine/School of Public Health, Kyoto University, Kyoto, Japan

**Keywords:** Electronic health record, Pragmatic trial, Mood disorder, Lithium carbonate, Randomized controlled trial

## Abstract

**Background:**

The weaknesses of classical explanatory randomized controlled trials (RCTs) include limited generalizability, high cost, and time burden. Pragmatic RCTs nested within electronic health records (EHRs) can be useful to overcome such limitations. Serum lithium monitoring has often been underutilized in real-world practice in Japan. This trial aims to evaluate the effectiveness of the EHR-nested reminder system for serum lithium level monitoring in the maintenance of therapeutic lithium concentration and in the improvement of the quality of care for patients on lithium maintenance therapy.

**Methods:**

The Kyoto Toyooka nested controlled trial of reminders (KONOTORI trial) is an EHR-nested, parallel-group, superiority, stratified, permuted block-randomized controlled trial. Screening, random allocation, reminder output, and outcome collection will be conducted automatically by the EHR-nested trial program. Patients with a mood disorder taking lithium carbonate for maintenance therapy will be randomly allocated to the two-step reminder system for serum lithium monitoring or to usual care. The primary outcome is the achievement of therapeutic serum lithium concentration between 0.4 and 1.0 mEq/L at 18 months after informed consent.

**Discussion:**

The KONOTORI trial uses EHRs to enable the efficient conduct of a pragmatic trial of the reminder system for lithium monitoring. This may contribute to improved quality of care for patients on lithium maintenance therapy.

**Trial registration:**

University Hospital Medical Information Network (UMIN) Clinical Trials Registry, UMIN000033633. Registered on 3 July 2018.

## Background

### Background and rationale

#### Limitations of classical “explanatory” randomized control trials and the potential of the electronic health record nested randomized control trial

Randomized control trials (RCTs) have been the gold standard to evaluate the benefits and harms of interventions. However, problems with the classical “explanatory” RCTs have been reported [1]. First, the generalizability of the results of explanatory RCTs is usually limited because only ideal patients are included owing to the RCT stringent eligibility criteria [2]. In addition, the recruitment of adequate numbers of participants is becoming increasingly difficult because of the expense and the time burden on clinicians [3].

Pragmatic trials have been proposed to increase the generalizability by conducting RCTs under routine clinical practice conditions rather than in specialized environments [4]. For the past few decades, electronic health records (EHRs) have been used widely for observational pharmacoepidemiology and clinical studies [5, 6]. Recently, some pragmatic RCTs, which are called EHR-nested RCTs, have used EHRs to assess the effectiveness of various interventions [7–11]. The EHR-nested RCT aims to increase the feasibility of RCTs by using an EHR to reduce trial costs, time burden, and human resources [12, 13]. The use of EHRs may allow pragmatic RCTs to be conducted in routine care settings. In addition, EHR-nested systems were shown to increase the referral and recruitment of participants in some studies [14, 15]. Although the benefits of EHR-nested RCTs have continued to emerge, most of these studies have been conducted in the USA and UK, and the feasibility of EHR-nested RCTs in other clinical environments is still uncertain [3, 9, 11, 16].

#### Mood disorder and lithium carbonate

The World Health Organization has reported that major depression is the third most common cause of years lost due to disability and the 16th most common cause of disability-adjusted life years [17]. Behind alcohol abuse, major depressive disorder is the second most frequent psychiatric disorder in Japan, with a lifetime prevalence of 5.7%. Bipolar disorder is rare compared with major depressive disorder; the lifetime prevalence of bipolar I disorder is 0.4% [18].

Lithium carbonate is a mood stabilizer used for maintenance treatment of bipolar disorder and recurrent unipolar depression [19, 20]. Owing to its narrow therapeutic range, regular serum lithium monitoring is strongly recommended, every week in the acute phase and every 3 months during the subsequent maintenance phase [21–23]. However, the rates of monitoring vary greatly in different countries, from 30% to 65% [24, 25]. In 2012, the Pharmaceutical and Medical Devices Agency (PMDA) in Japan warned against poor utilization of serum lithium monitoring, because 52% of patients who were prescribed lithium carbonate had not had their serum lithium monitored [26]. However, despite the regulatory alerts, a recent study revealed that serum lithium was monitored at least once a year in only 15% of patients [27]. After a substantial literature search, we identified no study of monitoring to improve the adherence of lithium monitoring.

### Objective

The purpose of the Kyoto Toyooka nested controlled trial of reminders (KONOTORI trial) is to examine whether the EHR-nested reminder system for serum lithium monitoring is feasible in routine clinical practice in Japan, and whether it can help to achieve therapeutic serum lithium concentration and improve outcomes for patients on lithium maintenance therapy.

## Methods/design

### Trial design

The trial is an open-label, parallel-group, single-center, superiority RCT to evaluate the effectiveness of an EHR-nested reminder system for serum lithium monitoring. The trial program in the EHR system automatically prompts for eligibility screening, conducts the random allocation, outputs reminders, and collects outcomes.

### Participants, interventions, and outcomes

#### Study setting

The trial is being conducted at the outpatient clinic of a psychiatry department at Toyooka hospital, a 518-bed tertiary care community hospital in Toyooka City, Hyogo, Japan, which has a population of 85,000. All physicians participating in the trial are psychiatrists. The trial program covers the entire EHR system in Toyooka Hospital but the reminder itself works only at the department of psychiatry.

### Participants

#### Eligibility criteria

Participants will be recruited in accordance with the eligibility criteria described below.

##### Inclusion criteria

The participant must fulfill all the following:

1. Age ≥ 18 years on the day of informed consent
2. Has recurrent major depression, bipolar I disorder, or bipolar II disorder according to the *Diagnostic and statistical manual of mental disorders, 5th edition* (DSM-5)
3. Has been taking lithium carbonate for 6 months or longer
4. Has been judged by the treating physician to need a prescription of lithium carbonate for the next 18 months

##### Exclusion criteria

The participant must not meet any of the following criteria:

1. Prescribed lithium carbonate for an indication other than mood disorders
2. A primary diagnosis of schizophrenia
3. Judged by the treating physician to have an imminent high risk of suicide
4. Suspected to have lithium intoxication
5. Women who are pregnant or breastfeeding
6. Cohabiting family members of study staff personnel
7. Inability to understand written Japanese
8. Contraindications to lithium carbonate
9. Participating in another clinical trial
10. Currently hospitalized
11. Terminal physical disease
12. No serum lithium concentration available within 7 days of informed consent
13. No appointment between 4 and 8 months after informed consent
14. Written informed consent is unavailable
15. Judged by the treating physician as inappropriate for participation

(criteria 12 and 13 may be confirmed after informed consent, but before randomization)

### Interventions

The trial program outputs two-step reminders of serum lithium monitoring to the treating physician as specified by the algorithm. When a participant in the intervention group visits the outpatient clinic between 4 and 8 months after the last lithium monitoring or at the study registration, reminder A will be sent to the treating physician. If the participant visits within 8 months after reminder A, reminder B will be sent. After reminder B has been sent and the participant visits the clinic thereafter, reminder A will be sent another time. No reminder will be sent if the participant receives lithium monitoring within 4 months, visits the outpatient clinic after 8 months or more, or if the participant is in the control group.

We anticipate that the two-step reminders will improve both the physician’s awareness and the participant’s adherence:

- Reminder A

The text of reminder A is as follows: “Please notify the participant of the need for a blood test for serum lithium level at the next outpatient visit. If the next visit will be 8 months or further from the previous blood test, please notify the participant of the need to conduct a blood test today. The participant and the treating physician can decide whether to conduct the blood test.”

- Reminder B

The text of reminder B is as follows: “Please notify the participant the need for a blood test for serum lithium level today. The participant and the treating physician can decide whether to conduct the blood test.”

#### Control

Participants in the control group receive usual care without reminders.

#### Concurrent treatment and concerns about contamination

There are no restrictions on concurrent treatments in the trial.

### Outcomes

At the first scheduled visit, between 18 and 24 months after informed consent, the program will output a reminder for the final evaluation. The treating physician will conduct a blood test within 7 days of the visit.

#### Primary outcome

The primary outcome is the achievement of therapeutic serum lithium concentration between 0.4 and 1.0 mEq/L at 18 months after informed consent. If a participant withdraws during the follow-up period or the result of the final blood test is not available, he/she will be regarded as not having achieved the primary outcome, because unavailability for the final serum lithium measurement strongly implicates that the patients is not adherent. However, the validity of this assumption will be tested in a sensitivity analysis using multiple imputation (see “Statistical analyses”).

#### Secondary outcomes

The secondary outcomes are as follows:

1. The number of blood tests for serum lithium concentration in the 18 months period after the date of informed consent.
2. Exacerbation of major depression or bipolar disorder in the 18-month period from the date of informed consent, defined by at least one of the following: hospitalization; increase in lithium dose; addition of antipsychotic drugs or mood stabilizer (valproic acid, carbamazepine, lamotrigine); or addition of or increase in use of antidepressants.
3. Proportion of days covered (PDC) of lithium carbonate prescription during the 18 months after informed consent [28].
4. Thyroid-stimulating hormone (TSH) ≥ 1.0 μIU/mL after 18 months.
5. Estimated glomerular filtration rate (eGFR) < 60 mL/min per 1.73 m^2^ after 18 months.

### Participant timeline

**Fig. 1:**
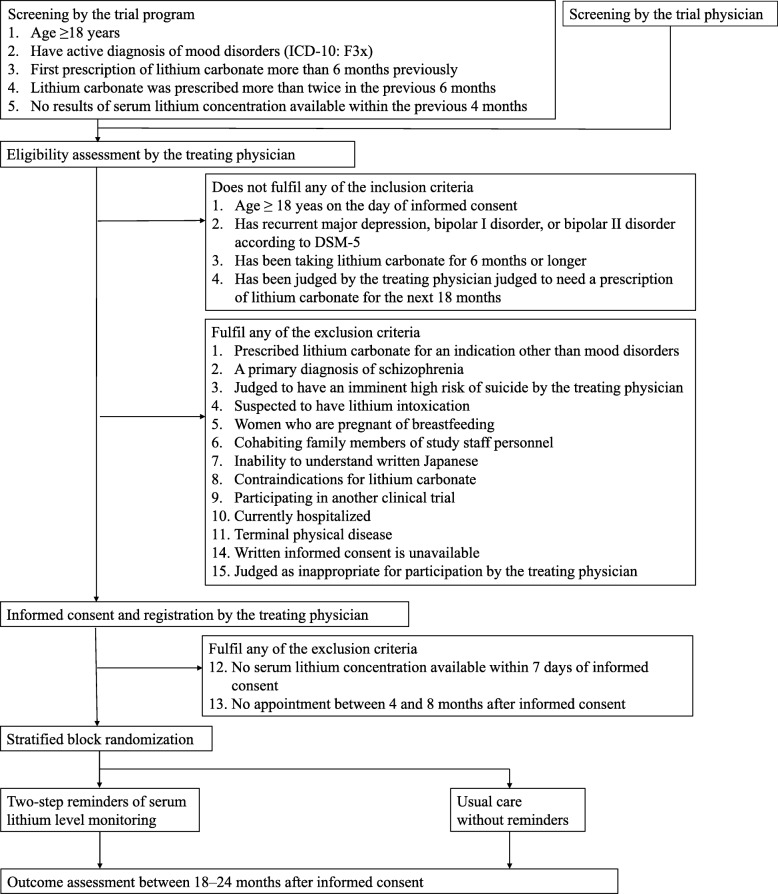
Flow diagram of the trial. Exclusion criteria 12 and 13 are confirmed after informed consent, but before randomization. ICD: *International classification of disease*, DSM: *Diagnostic and Statistical Manual of Mental Disorders*

The timeline for the study participants is shown in Fig. 1 and the enrollment, intervention and assessment schedule is shown in Table 1.

**Table 1:** Schedule of the enrollment, intervention, and assessments

	Study periods							
		Enrollment	Allocation	Post-allocation				Close-out
	Timepoints	T_-1_	T_0_	T_1-A_	T_1-B_	T_2-A_	T_2-B_	T_x_^e^
Trial program	Enrollment							
	Screening	X						
	Randomization^a^		X					
	Intervention							
	Reminder A			X		X		
	Reminder B				X		X	
	Assessments							
	Baseline characteristics^b^	X						
Treating physician	Enrollment							
	PRIME-MD^c^	X						
	Eligibility assessment	X						
	Informed consent	X						
	Assessments							
	Lithium concentration^d^	X						X
	Liver function	X						X
	Kidney function	X						X
	Thyroid function							X

*PRIME-MD* Primary Care Evaluation of Mental Disorders

^a^Stratified block randomization will be conducted if the candidate has a measurement of serum lithium within 7 days of giving informed consent and an appointment between 4 and 8 months after giving informed consent

^b^Basic information includes gender, age, and concomitant medication

^c^The candidate will be classified as having major depression, bipolar I disorder, or bipolar II disorder

^d^Serum lithium will be classified into three groups (between 0.4 mEq/L and 1.0 mEq/L inclusive; < 0.4 mEq/L; or > 1.0 mEq/L)

^e^The first scheduled visit is between 18 and 24 months after informed consent

#### Screening by the trial program

The trial program automatically screens candidates who fulfill all of the following criteria from the EHR every morning:

1. Age ≥ 18 years
2. Have active diagnostic codes of mood disorders (F30.x, F31.x, F32.x, F33.x, F34.x, F38.x, and F39.x) according to the *International classification of diseases* (ICD), 10th revision
3. First prescription of lithium carbonate more than 6 months previously
4. Lithium carbonate was prescribed more than twice in the previous 6 months
5. No serum lithium concentration measurement available within the previous 4 months

The algorithm that combined lithium prescription more than twice and the prescription period longer than 180 days ensure lithium continuation over 180 days, because the maximum prescription period is 90 days in Japan. The participant will not be screened if he/she has been already registered as “not eligible”, “refused participation”, or “withdrawal after participation”.

#### Eligibility check and registration

Primary Care Evaluation of Mental Disorders (PRIME-MD) is a semi-structured diagnostic tool to assist primary care physicians in the diagnosis of common mental disorders based on the DSM-5 [29]. The treating physician conducts the PRIME-MD and confirms the diagnosis of depression, bipolar I, or bipolar II. If the candidate meets the eligibility criteria and written informed consent is obtained, the treating physician registers the participant through the EHR. If the candidate is not eligible, refuses participation, or withdraws after participation, the treating physician enters the status through EHR. The treating physician will conduct a baseline blood test within 7 days of the patient giving informed consent. If the candidate does not receive the baseline blood test or a measurement of serum lithium is not available within 7 days, he/she will be excluded from the study.

### Sample size calculation

We estimated the sample size using Real World Data (RWD) database (Health, Clinic, Education, Information Evaluation Institute/Real World Data, Co., Ltd). Toyooka Hospital provides anonymized patient data to the database, which includes about 19 million patients’ data from 170 institutions in Japan. In RWD database, 1464 patients were prescribed lithium carbonate in a 6-month period. Of those, serum lithium was maintained between 0.4 mEq/L and 1.0 mEq/L in 818 (55.9%) patients. Although RWD database includes patients from Toyooka Hospital, the number of patients in Toyooka Hospital is unclear because numbers of patients in each hospital were not provided to the researcher.

In the trial, we assumed that 80% of participants in the intervention group and 55% of patients in the control group will achieve the goal. As 54 participants are needed for each group, assuming a dropout rate of 10%, a total of 120 participants is required to detect this difference with a type I error of 5% and a type II error of 10%, using the two-sided chi-square test.

### Assignment of intervention

#### Randomization

The participants will be stratified into nine strata in accordance with the following two conditions in EHR, and automatically randomized by the trial program at their first scheduled visit between 4 and 8 months after informed consent, using permuted block randomization [30].

- Diagnosis based on PRIME-MD (major depression, bipolar I disorder, or bipolar II disorder)
- Serum lithium (between 0.4 mEq/L and 1.0 mEq/L; < 0.4 mEq/L; or > 1.0 mEq/L)

#### Allocation sequence generation and concealment

Random allocation sequence is generated by an independent trial statistician (ST) but will not be notified to other researchers and participants. The allocation sequence is stored in the trial program and then concealed from other researchers.

### Masking

Allocation status will not be masked to the treating physician and the participant. Allocation status will be masked to the data manager, the trial statistician, and the steering committee until the steering committee finalizes the interpretation report with masked data.

### Stopping rules for participants and trial

#### Stopping trial intervention

If the participant meets any of the following conditions, the treating physician will stop the trial intervention and record the date and the reason. However, the participant is not considered to have dropped out of the study at this stage and will receive the protocol assessments:

1. The participant wishes to withdraw from the trial intervention and/or treatment with lithium carbonate
2. The treating physician judges that it is difficult to continue the trial intervention and/or lithium carbonate owing to the occurrence of serious adverse events
3. The treating physician judges that the trial intervention and/or lithium carbonate should be stopped for clinical reasons other than serious adverse events
4. The Steering Committee judges that the trial intervention or lithium carbonate should be stopped for any reason

#### Stopping assessment

If the participant withdraws their consent to the protocol assessments, the participant will be excluded from analyses regardless of whether he/she continues the trial intervention and/or lithium carbonate.

The participant will be excluded from analyses if, after informed consent, it is found that the participant did not meet the eligibility criteria. In this case, the participant will not be considered to have stopped the trial intervention or the protocol assessments.

#### Stopping criteria for the trial

The Steering Committee will stop the trial upon advice or orders from the Ethics Committee if either of the following conditions are met:

1. Any serious problem in the quality, safety, and efficacy of the trial intervention and/or lithium carbonate
2. When the Ethics Committee issues and order for changes to the protocol that are unacceptable to the researchers

The decision to stop the trial, and the reason for this, will be reported to the Ethics Committee and research staff as soon as possible.

### Data collection and management

All the patient data are stored in the EHR and the password-protected trial program on a local server automatically stores the information on screening, randomization, and the intervention. After trial completion, researchers manually extract anonymized patient data from the trial program and EHR, check the data quality, and finalize the data set.

### Statistical analysis

All analyses will be conducted based on the intention-to-treat principle. The primary outcome will be analyzed from the full analysis set using logistic regression and reported with the odds ratio, *p* value, and 95% confidence interval. The model includes the primary diagnosis based on PRIME-MD (major depression, bipolar I disorder, or bipolar II disorder) and baseline serum lithium (0.4–1.0 mEq/L; < 0.4 mEq/L; or > 1.0 mEq/L). The validity of our assumption equating missing the final serum lithium measurement with not achieving the primary outcome will be examined in sensitivity analysis using multiple imputation. Secondary analyses and subgroup analyses will be conducted to supplement the primary analysis and explore a deeper understanding of the study questions. As secondary and subgroup analyses are exploratory, we will not adjust the significance level for multiple testing. All statistical tests will be two-sided and *p* values <0.05 will be considered statistically significant. The details of the statistical analysis will be decided within the statistical analysis plan, which will be reviewed and approved by the trial statistician and become publicly available before outcomes of the last participant are registered and therefore while the treatment allocation is still unknown. No interim analyses will be conducted in this trial.

### Monitoring

#### Data monitoring

The data manager, in conjunction with the clinical management team, conducts weekly central monitoring and annual on-site monitoring in the trial. In the central monitoring, we monitor the number of patients screened, eligible, included, and allocated by the weekly central monitoring. In addition, the data manager conducts on-site monitoring every 6 months after registration of the first case, to check the logistics, such as the informed consent document.

### Site audit

No formal site auditing will be conducted, because the trial comprises only a minimally invasive intervention.

### Harms

Adverse events are defined as follows: any undesirable or unintended signs including anomalies in clinical laboratory evaluations, symptoms, or diseases among participants, regardless of whether there is a causal relationship with the treatment. The treating physician will provide and/or arrange appropriate treatments, including hospital admission if necessary. Because adverse events, including toxicities of lithium carbonate, are monitored as part of routine care, study-specific monitoring will not be required.

When a serious adverse event occurs, the treating physician must take all the necessary and appropriate measures to ensure the safety of the participant. The treating physician must also notify the principal investigator and report to the chief study investigator at Toyooka Hospital within 24 h. The principal investigator must notify the Ethics Committee of the Graduate School of Medicine, Kyoto University Graduate School of Medicine within 72 h. The chief study investigator at Toyooka Hospital must report to its own Institutional Review Board (IRB) and, if it concerns an unforeseeable serious adverse event, must report to the Ministry of Health, Labour and Welfare (MHLW). A serious adverse event is defined as one that may lead to death or to continuous severe impairment, depending on the patient’s conditions and circumstances.

### Foreseeable adverse events

No adverse events are foreseeable for the trial intervention itself, except for the potential burden and complications of blood tests. All drugs, including lithium carbonate, are within the approved dosages and indications according to the MHLW.

The foreseeable side effects of lithium carbonate, as described in the package insert, are as follows:

Major side effects: Tremor (4.6%), dry mouth (2.4%), and diarrhea (1.2%). Serious side effects: Lithium intoxication (unknown frequency); malignant syndrome (unknown frequency); sick sinus syndrome and bradycardia (unknown frequency); nephrotic diabetes insipidus (unknown frequency); acute kidney injury, interstitial nephritis, and nephrotic syndrome (unknown frequency); hypothyroidism and thyroiditis (unknown frequency); hyperparathyroidism (frequency unknown); and dementia and impaired consciousness (frequency unknown).

### Ethics and dissemination

#### Adherence to the study protocol

All researchers participating in the trial will place the safety and human rights of the participants at the highest priority and will adhere to the study protocol, as long as it does not compromise their safety and human rights.

#### Regulations to be adhered to

All researchers participating in the trial will abide by the Declaration of Helsinki and its amendments, and by the Ethical guidelines for medical and health research involving human subjects (2017 revision, Ministry of Education, Culture, Sports, Science and Technology, and MHLW).

### Approval by the Institutional Review Boards

The trial has been approved by the Ethics Committee of the Kyoto University Graduate School of Medicine (registration number: C1401) and the Institutional Review Boards of Toyooka Hospital (registration number: 180).

### Protocol amendments

Any changes to the study protocol will be reported to the Ethics Committee of the Kyoto University Graduate School of Medicine for approval. If approval is granted, approval for the study protocol change will subsequently be sought from the Institutional Review Board of Toyooka Hospital. Changes will then be disseminated to the research staff, and, where necessary to the study participants.

### Informed consent

#### Procedures for informed consent

The treating physician must make sure that the participant has understood the contents of the trial and must obtain written informed consent from the participant. The treating physician must seek repeat consent when the research protocol is revised, and any invasion or possible disadvantage is applied to the participant who has already consented.

### Confidentiality

All researchers and contractors of the trial must strictly manage participants’ personal information in adherence with the Ethics Guideline for Clinical Research and Act on the Protection of Personal Information. The data manager will delete site-specific patient IDs and add study-specific patient IDs at the time of data retrieval from Toyooka Hospital. Before the data manager retrieves the data, the data manager and the chief study investigator at Toyooka Hospital will ensure the data anonymity. The chief study investigator will keep the correspondence table up to date, including both the study-specific and the site-specific patient IDs at Toyooka Hospital. The data manager and the trial statistician will have access only to anonymized data and will not have access to the participants’ personal information. The media will be stored in a locked cabinet in the office of the Department of Pharmacoepidemiology, School of Public Health, Department of Pharmacoepidemiology, Graduate School of Medicine/School of Public Health, Kyoto University, for 10 years after the publication of the main results and will then be discarded.

### Access to the data

All members of the Steering Committee will have full access to all the final dataset.

### Ancillary and post-trial care

All participants will receive standard care during and after the trial.

#### Exemption of medical expenses and rewards

As all the diagnostic tests and treatments in the trial are within the MHLW-approved dosage and administration, medical expenses will be covered in the usual way through the participant’s health insurance and copayment. Participants will not receive any reward or exemption for any tests or treatments.

#### Compensation for adverse events

As all the diagnostic tests and treatments in the trial are conducted in accordance with the indications and dosage approved by the MHLW, the costs of care for any adverse events will be covered by the participant’s health insurance and copayment. We will not contract private health insurance for clinical research because the trial involves only a minimally invasive intervention.

### Dissemination policy

The protocol will be published in an English-language academic journal and presented at a scientific conference. The synopsis will be available from the website of the Department of Health Promotion and Human Behavior, Department of Pharmacoepidemiology, Graduate School of Medicine/School of Public Health, Kyoto University, and Toyooka Hospital. The trial results will be disseminated through academic journals and conference presentations. A summary of study results will be disseminated on the website listed above for dissemination to trial participants.

Authorship for the primary and secondary results will be determined by all members of the Steering Committee. If the chief study investigator, participating physicians, and other members of the Steering Committee do not appear as co-authors, they will be listed at the end of the article. Such authors may be listed as co-authors in some journals, but not in others.

After the publication of the main findings, we will register the anonymized dataset in UMIN-Individual Case Data Repository (ICDR) (http://www.umin.ac.jp/icdr/index-j.html). Only researchers who are certified by the Steering Committee will be allowed access the data.

## Discussion

KONOTORI is an EHR-nested, parallel-group, superiority RCT examining the effectiveness of the reminder system for serum lithium monitoring. This trial includes some prominent characteristics.

First, as mentioned previously, the trial program embedded with the EHR automatically prompts for screening, conducts random allocation, outputs reminders, and collects data without extra cost and time burden in everyday clinical practice. Embi et al. demonstrated a 10-fold increase in referral rate and the doubling of recruitment with an EHR-nested recruiting system [14]. Recently, several EHR-nested RCTs have been implemented for the assessment of the effectiveness of various interventions, such as alerts on kidney injury, warnings for harmful drug-drug interactions, laboratory test abnormalities, personalized antibiotics prescription feedback, or dosing errors [7, 9, 11]. The REDUCE trial is ongoing to reduce unnecessary antibiotic prescriptions for respiratory tract infections in general practice, with a monthly feedback, educational, decision support tool, and webinar using EHR [8]. Similarly, the Standard and new antiepileptic drugs II (SANAD II) trial is assessing the clinical and cost effectiveness of antiepileptic treatments for patients with newly diagnosed epilepsy [10]. However, the feasibility of EHR-nested RCTs in Japan is unclear, because the aforementioned trials were conducted in Western countries, mostly in the USA and UK. The KONOTORI trial will be the first EHR-nested RCT not conducted in a Western country.

Second, in our sample size calculation with an EHR-based administrative database including Toyooka hospital, about 45% of patients had serum lithium of < 0.4 mEq/L or > 1.0 mEq/L. We assumed that the infrequent monitoring may partially have caused such deviation. Then, we expect that more frequent monitoring triggered by the reminders may be useful to maintain serum lithium within the appropriate range and achieve subsequent clinical outcomes. The two-step reminders (A and B) are to remind not only the physicians but also the patient. The reminders may, through the increased frequency of the reminder to the physician, the increased awareness on the part of the physicians, the increased recommendation from the physician to the patients based on such reminders, and/or through increased adherence on the part of the patients resulting from such interactions with the physician, help achieve the primary outcome of the therapeutic serum concentration. Then, it is not feasible to mask the patient, because we remind not only the physician to increase the frequency but also the patient to improve adherence. If the automated reminder system is found to be effective for achieving the appropriate serum lithium level in patients taking lithium carbonate in this study, this may contribute directly to improvement in the quality of clinical practice, because its implementation does not require additional effort by clinicians. On the one hand, the trial was so designed to pragmatically examine the net effect of this procedure and not to explore the mechanisms by administering additional questionnaires to the physicians and the patients. This may be a weakness but also a strength in increasing the practicality of the trial and also the generalizability of the final findings through facilitated recruitment.

Finally, both the EHR-nested RCT scheme and the EHR-nested automated reminder system may be applicable in medical specialties other than psychiatry. Overall, if the EHR-nested trial is successful, it can be expected to contribute to the so-called learning healthcare system, where every clinical encounter in routine care can provide an opportunity to expand the evidence base for future health care [31] (Additional file 1).

### Limitations

We acknowledge some difficulties in the trial. First, “alert fatigue” is possible; when treating physicians are exposed to too many clinical decision support alerts, they may stop responding to the alerts [32]. However, this risk will be relatively low in the present trial, because, in principle, the frequency of reminders is once every 6 months.

Second, because the study will be conducted in a single tertiary care center and randomization will be conducted based on individual patients rather than cluster randomization, the number of blood tests and level of patient adherence may increase in the control group as well as in the intervention group through contamination among the participating physicians. Because the trial is conducted at a single facility, it is difficult to avoid such contamination; however, this is expected to result in underestimation rather than overestimation of the effectiveness of the EHR-reminder system. In future studies, the inclusion of multicenter and cluster randomization may be necessary to minimize this risk.

Third, a clinical endpoint such as exacerbation of mood disorders is more appropriate as the primary outcome than surrogate markers such as serum lithium in a pragmatic trial. However, the expected number of exacerbations is not large within the study period and therefore we do not have enough statistical power to detect the difference in a true endpoint in the trial. On the other hand, another objective of the trial is proof of concept of an EHR-nested RCT in the Japanese setting. A surrogate outcome may be sufficient for the purpose. Then, we chose serum lithium instead of the number of blood tests, one of the secondary outcomes, because it was considered the most clinically relevant among surrogate outcomes.

Fourth, the sample size with the drop-out rate of 10% and the effect size of 25% may be too optimistic. Higher drop-out rates of 25–60% have been reported in some psychiatry trials compared to a general drop-out rate of 7% [33–35]. However, these studies were largely different from ours because most of them were derived from acute-phase trials with placebo control or active comparator. Our study includes patients after a 6-month run-in period, who are expected to continue taking lithium carbonate over 18 months. In addition, the study is a single-center study, and most moderate-to-severely affected patients are followed at Toyooka hospital because it is the only institution in the region to provide inpatient psychiatric care. Furthermore, the estimated risk difference of 25% is also arbitrary, although evidence on reminders or alert systems for lithium monitoring is lacking.

Fifth, non-serious adverse events may be underreported because we monitor non-serious adverse events from the spontaneous reporting of each treating physician and the final blood test. However, underreporting is not likely for serious adverse events because researchers are obliged to report all the serious adverse events to the MHLW under the Ethical guidelines for medical and health research involving human subjects (2017 revision, Ministry of Education, Culture, Sports, Science and Technology, and MHLW).

Sixth, if serum lithium is not tested despite the reminder, we are unable to determine whether the physician or the patient was responsible for not conducting the blood test.

After consideration of all these aspects, we hope that the KONOTORI trial and the EHR-nested RCTs will prove to be successful models for future clinicians, researchers, and decision-makers in the learning healthcare system [31].

## Trial status

Participant recruitment was started in November 2018 and is ongoing at the time of submission of the protocol paper. We have recruited 101 patients since 1 November 2018 to 11 September 2019. We estimate that recruitment will be complete by 31 March 2020.

## Supplementary information

**Additional file 1.** SPIRIT 2013 Checklist: Recommended items to address in a clinical trial protocol and related documents.

## Data Availability

After the publication of the main findings, we will register the anonymized data set in UMIN-Individual Case Data Repository (ICDR) (http://www.umin.ac.jp/icdr/index-j.html). Only researchers who are certified by the Steering Committee will be allowed access the data.

## Abbreviations

DSM: Diagnostic and Statistical Manual of Mental Disorders
EHR: Electronic health record
ICD: International Classification of Diseases
ICDR: Individual Case Data Repository
KONOTORI: Kyoto Toyooka nested controlled trial of reminders
MHLW: Ministry of Health, Labour and Welfare
PDC: Proportion of days covered
PMDA: Pharmaceutical and medical devices agency
PRIME-MD: Primary Care Evaluation of Mental Disorders
RCT: Randomized control trial
SANAD: Standard and new antiepileptic drugs
UMIN: University Hospital Medical Information Network
